# Theoretical derivation and clinical dose-response quantification of a unified multi-activation (UMA) model of cell survival from a logistic equation

**DOI:** 10.1259/bjro.20210040

**Published:** 2021-11-04

**Authors:** Shidong Li

**Affiliations:** ^1^ Department of Radiation Oncology, Temple University Hospital, Philadelphia, PA, USA

## Abstract

**Objective:**

To theoretically derive a unified multiactivation (UMA) model of cell survival after ionising radiation that can accurately assess doses and responses in radiotherapy and X-ray imaging.

**Methods:**

A unified formula with only two parameters in fitting of a cell survival curve (CSC) is first derived from an assumption that radiation-activated cell death pathways compose the first- and second-order reaction kinetics. A logit linear regression of CSC data is used for precise determination of the two model parameters. Intrinsic radiosensitivity, biologically effective dose (BED), equivalent dose to the traditional 2 Gy fractions (EQD2), tumour control probability, normal-tissue complication probability, BED_50_ and steepness (Γ50) at 50% of tumour control probability (or normal-tissue complication probability) are analytical functions of the model and treatment (or imaging) parameters.

**Results:**

The UMA model has almost perfectly fit typical CSCs over the entire dose range with R^2^≥0.99. Estimated quantities for stereotactic body radiotherapy of early stage lung cancer and the skin reactions from X-ray imaging agree with clinical results.

**Conclusion:**

The proposed UMA model has theoretically resolved the catastrophes of the zero slope at zero dose for multiple target model and the bending curve at high dose for the linear quadratic model. More importantly, it analytically predicts dose–responses to various dose–fraction schemes in radiotherapy and to low dose X-ray imaging based on these preclinical CSCs.

**Advances in knowledge:**

The discovery of a unified formula of CSC over the entire dose range may reveal a common mechanism of the first- and second-order reaction kinetics among multiple CD pathways activated by ionising radiation at various dose levels.

## Introduction

For over a century, radiobiologists, medical physicists, and clinicians who use ionising radiation for medical imaging and therapy, have been searching for a simple formula to accurately describe the intrinsic radiosensitivity of cell lines to various doses or the cell survival curves (CSCs). A single-hit multitarget (MT) model by Lea et al^1,2^ has cell survival fraction $S\left(D\right)=1-{\left(1-e^{-D/D_{o}}\right)}^{n}$ , where *n*, *D* and *D_o_* are the number of targets in a cell, dose and the 37% dose slope on the exponentially straight portion of a typical CSC at high doses, respectively. A multiple-hit MT model by Puck & Marcus^3^ has $S\left(D\right)=1-{\left[1-\sum_{j=0}^{m-1}e^{\frac{-D}{D_{o}}}{\left(\frac{D}{D_{o}}\right)}^{j}/j!\right]}^{n}$ to describe the shoulder of the HeLa CSC to X-rays, where m is the number of hits per target site. Both the single-hit and multiple-hit MT models have the zero slope at the zero dose that conflicts with constant slopes of observed CSCs to high linear energy transfer (LET) radiations. A two-component MT model by Bender & Gooch^4^ has $S\left(D\right)=e^{-k_{1}D}\left[1-{\left(1-e^{-k_{2}D}\right)}^{n}\right]$ to define a non-zero initial slope by the third parameter *k_1_* which has also complicated its theoretical interpretation and clinical application. A linear-quadratic (LQ) model^5^ of $y=-ln\left(S\right)=\alpha D+\beta D^{2}$ for the yield of chromosomal aberrations has a theoretical derivation by Kellerer and Rossi^6^ to correlate the linear and quadratic terms to the intra- and intertrack actions, respectively. The target size is estimated by Zaider and Rossi^7^ to be the size of DNA-helix. A molecular theory of cell survival by Chadwick and Leenhouts^8^ has also concluded “that the induction of DNA double strand breaks (DSB) should be linear-quadratic”. The LQ model became the clinical standard in assessing radiosensitivity and radiocurability in radiology and radiotherapy with its conspicuous advantages of simple formulation and plausible interpretations but the bending curve discrepancy from the straight portion of many CSCs at high doses has never been forgotten. An early attempt is the lethal and potential lethal (LPL) damage repairing model by Curtis^9^ to extrapolate a CSC as an exponentially straight line at high dose but its four parameters and five assumptions prohibits it from clinical usage. Recently, advances of hypofractionated radiotherapy have encouraged investigators to develop an effectively linear-quadratic-linear (LQL) model by methods of: (1) changing the β parameter as a function of time and repairing-rate constants^10^; (2) adding a function of dose shift and constants^11^; or (3) combining the LQ model at the low-dose domain, the MT model at the high-dose domain, and a transition dose point between the two dose domains for a universal survival curve (USC) model.^12,13^ However, these LQL models are either inconvenient to use clinically as they involve complicated functions and difficult to explain theoretically for mechanism changes at different dose levels, or potentially uncertain to correlate outcomes with more parameters. A unified model of CSCs over the entire dose range with a common mechanism is desired for clinical applications and theoretical extensions to very low and high doses that have perplexed us for a long time.^14,15^

## Methods and materials

### Derivation of a unified formula

There are millions of possible chemical reactions from thousands of different molecules in a cell but only these sped-up by available protein enzymes takes place at measureable rates. Radiation-induced DNA damages and free radicals may trigger some biochemical reactions that would lead cell death (CD) through some CD pathways (or cell dying processes) with distinct morphologies or molecular mechanisms.^16^ The radiation-induced reactions are assumed to be the first- and second-order reaction kinetics in the cellular scale. Based on the evidences that ionising radiation produces free radicals and DNA damages at a rate proportional to the dose rate, *e.g.* ~40 DSBs/Gy from X-ray radiation, the first-order reactions would depopulate the cells of interest at a rate of $\gamma \dot{D}N$ , where *γ,* $\dot{D}$ and N are the apparent first-order-reaction activation constant per unit of dose, dose rate and number of cells in the region of interest, respectively. The exponentially straight CSC to α particles is an example of CD through the first-order cellular kinetics. The second-order reactions would depopulate the cells at a rate of $-dS/S^{2}=\delta NodD$ , where *δ* is the apparent second-order-reaction activation constant per unit of N per unit of dose. The second-order kinetics has dominated the renaturation of isolated fragments from mammalian DNA.^17^ The combination of the first- and second-order reactions in the dose domain results

(1) $$-dN/dt=\overset{˙}{D}\left(\gamma N+\delta N^{2}\right)⟹dN=-\gamma \left[1+N/\left(\gamma /\delta \right)\right]NdD$$

where the ratio of γ/δ is the “carrying capacity” in the dose domain in unit of N. Eq. (1) is similar to the logistic growth of cells^18^ but converted the CD in the dose domain.

**Figure 1.**
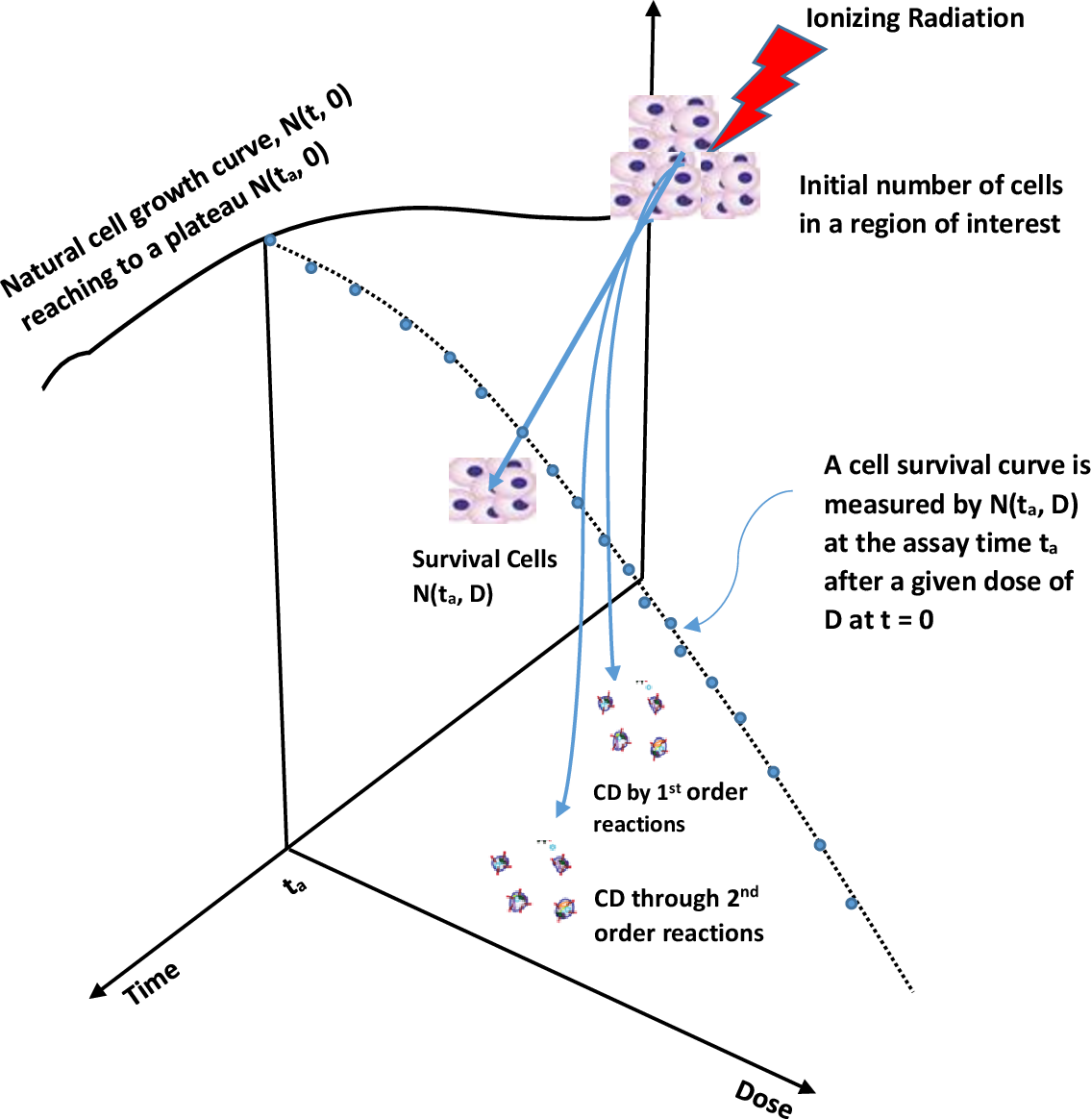
A sketch of *in-vivo* cell growth and cell survival curve (CSC) with the dynamic changes of cells (or colonies) in the time and dose domains. A natural cell growth curve is first observed by the number of survival cells (or colonies) in time without initial radiation. An assay at the assay time t_a_ on the plateau of the cell growth curve provides a stable number of cells, *N(t_a_, 0)=N_o_*, that is the reference number of the cells (or colonies) in measuring the CSC in the dose domain.

Figure 1 illustrates how an *in-vivo* CSC is obtained with the dynamics of cells (or colonies) in the time and dose domains. A natural cell growth curve is the plot of numbers of survival cells (or colonies) in time with no initial radiation. An assay time *t_a_* is selected on the plateau of the cell growth curve so that a stable number of cells, *N(t_a_, 0)=N_o_*, provides a reliable reference number of the cells (or colonies) for measuring the CSC in the dose domain. Observed number of survival cells (or colonies) at the assay time for a cohort of cells with an initial dose of D gives the survival fraction of *S(D)=N(t_a_, (**D**)/N_o_*. Eq. (1) in term of *S* has a general solution (Supplementary Material 1) of

(2) $$S\left(D\right)=\frac{n}{e^{\gamma D}-1+n}$$

where $n=\frac{\gamma }{\gamma +\delta N_{o}}$ is the ratio of the first-order reaction rate of $\gamma \dot{D}$ to the total reaction rate of ( $\gamma +\delta N_{o}\dot{D}$ ). The γ, δ and *n* are assumed to be dose-independent in the derivation.

A modified logit linear regression is useful to precisely determine the parameters *γ* and *n* in the unified formula. A parameterised logit function of $ln\left[\frac{S}{A-S}\right]=\gamma_{o}-\gamma D$ would transform all data points of a CSC into a straight line by iteratively adjusting the parameter of A. The best model fitting should have the coefficient of determination, R^2^, being closest up to one, and there would be no large residual of data points to the straight line except for outliers with large experimental errors. The logit-linear regressions with and without suspected outliers would ensure that the removal of the outliers had not caused major changes in the analysis for the estimation of the A, $\gamma_{o}$ and γ . The final results define the γ and $n=A∙e^{\gamma_{o}}$ .

### Model interpretations and definition of intrinsic radiosensitivity

The unified formula of Eq. (2) represents a CSC over the entire dose range that is derived from the apparent first- and second-order reaction kinetics for the cells of interest undergoing through multiple CD pathways activated by ionising radiation. Thus, it is a unified multiactivation (UMA) model. A positive γ would result a net CD (*S* < 1) from the first-order reactions while a negative γ may result a net cell growth (*S* > 1) or CD (*S* < 1) depending upon the *n*. There are five mathematical deductions for variations of *n*.

Deduction #1: n§amp;lt;0 . Formula (A4) of $n=S(e^{\gamma D}-1)/\left(1-S\right)$ allows only γ§amp;lt;0 and $S\in \left(\frac{n}{n-1},1\right)§amp;lt;1$ since the other possible solution of γ§amp;gt;0 and S§amp;gt;1 would contradict with *S* < 1 at large doses. This is a situation of ${\delta N}_{o}§amp;gt;-\gamma$ for a negative n in formula (A3), *i.e*. the CD from the second-order reactions overplays the cell growth from the first-order reactions.

Deduction #2: n=0 . the only non-trivial solution is *δ ≠ 0* and *γ = 0* for the second-order reactions only and Eq. (1) is replaced by *-dS/S^2^ = δN_o_dD* which has a solution of *S = 1/(δN_o_D + 1*) to satisfy the initial condition of *S(0) =* 1 with *δ > 0* to avoid any negative *S at* $D§amp;gt;\frac{-1}{\delta N_{o}}$ .

Deduction #3: $n\in \left(0,1\right)$ . Formula (A5) $\frac{\gamma }{\delta N_{o}}=\frac{n}{1-n}§amp;gt;0$ requires that γ and δ have the same sign. A negative γ with $n\in \left(0,1\right)$ is impossible to yield a negative survival of $S=\frac{n}{n-1}§amp;lt;0$ at any large doses. Positive γ with $n\in \left(0,1\right)$ have been obtained for typical CSCs with inverted shoulders. Hyperfractionated radiotherapy is desired for tumour cell lines with inverted shoulders.^19^

Deduction #4: n=1 . Eq. (2) is simplified as $S=e^{-\gamma D}with\frac{\gamma }{\delta N_{o}}=\frac{n}{1-n}\to \infty$ virtually for δ=0 . That is CD or cell growth from the first-order reactions only.

Deduction #5: n§amp;gt;1 . Formula (A5) $\frac{\gamma }{\delta N_{o}}=\frac{n}{1-n}§lt;-1$ requires that γ and δ have opposite signs. Typical tumour CSCs to X-rays have γ > 0 for CD through the first-order reactions and δ < 0 for cell growth or damage repairing through the second-order reactions. If there were a γ < 0 for cell growth from the first-order reactions and δ > 0 for CD from the second-order reactions, there would be a net cell growth with $S\in \left(1,\frac{n}{n-1}\right)§amp;gt;1$ .

In summary, excluding the initial condition of *S(0) = 1*, γ > 0 exists at *n* > 0 with S < 1; γ = 0 exists at *n* = 0 with *δ > 0* and *S = 1/(δN_o_D + 1*) < 1; γ < 0 mayt exist at *n* < 0 with $S\in \left(\frac{n}{n-1},1\right)§amp;lt;1$ , *n* >1 with $S\in \left(1,\frac{n}{n-1}\right)§amp;gt;1$ , or *n* = 1 with $S=e^{-\gamma D}§amp;gt;1$ . The UMA model predicts that it is possible for *S > 1* with γ < 0 and n≥1 at all dose levels of a CSC. But *S* > 1 has only been observed on some low dose points of a few CSCs and the UMA model fitting of these CSCs have resulted γ > 0 and the regression residuals on these low doses are within their experimental errors. Thus, positive γ is presumably used for all clinical applications of the UMA model.

The unified formula can analytically quantify the intrinsic radiosensitivity (RS) of any cell lines from the negative derivative of natural log survival to the dose as

(3) $$RS\left(D\right)=\frac{-dLn\left(S\right)}{dD}=\frac{\gamma }{n}Se^{\gamma D}=\frac{\gamma }{1+\left(n-1\right)e^{-\gamma D}}$$

The initial RS at the zero dose is γ/*n*, corresponding to the α parameter in the LQ model. At a very low dose of γD≪1 , RS approximates to $\gamma /\left[n+\left(1-n\right)\gamma D\right]$ . At a high dose of γD≫1 , RS approximates to the γ and cell survival approximates the straight line of $S\approx ne^{\gamma D}$ that is the same as the MT model with γ = 1/D_o_ but the γ and *n* are theoretically the first-order-reaction activation constant and the ratio of the first-order-reaction rate to the total reaction rate, respectively. The UMA model describes CSCs over the entire dose range with one mechanism and have resolved the catastrophes of zero slope at zero dose from MT models and of the bending curve at high dose from LQ model. Since RS changes with dose, it is reasonable to explore the new UMA model for global radiosensitivity (GRS) of a cell line to a type of radiation. For which, the reciprocal of the mean inactivation dose introduced by Fertil et al^20^ is applied for

(4) $$GRS=1/\int_{0}^{\infty }S\left(D\right)dD=1/\frac{D-\gamma ln\left(e^{\gamma D}+n-1\right)}{\left(n-1\right)}{|}_{0}^{\infty }=\frac{\gamma \left(n-1\right)}{nLn\left(n\right)}\;if\;n§gt;0$$

### Analytical predictions of clinically interested doses and responses

Based on recovery of RS mostly within the first few hours in delay plate and split dose experiments, the cell survival at the end of a course of D-Gy m-fractions with enough time between fractions for essentially complete repair of sublethal damage is clinically approximated by the multiplication of a fraction dose survival, *i.e*. *S(m, D*)=*S(D)^m^*. A biologically effective dose (BED) is defined by a single dose with the same survival of the course (Supplementary Material 1) as

(5) $$BED\left(m,D\right)=ln\left[1-n+{\left(e^{\gamma D}-1+n\right)}^{m}/n^{m-1}\right]/\gamma$$

The dose equivalent to 2 Gy fractions (EQD2) has been previously derived^19^ as

(6) $$EQD2\left(m,D\right)=2Gy∙m\frac{ln\left(e^{\gamma D}-1+n\right)-ln\left(n\right)}{ln\left(e^{\gamma ∙2Gy}-1+n\right)-ln\left(n\right)}$$

For a tumour or an organ consisting heterogeneous cell lines, the dominate radioresistant cell line to the tumour and radiosensitive cell line to the organs at risk are selected for computing the tumour control probability (TCP) and normal tissue complication probability (NTCP) at a follow-up time, respectively. Poisson distribution to approximate the binomial distribution of death or survival of cells gives

(7) $$TCP\vee NTCP\left(N_{o},m,D\right)=e^{-N_{o}S\left(m,D\right)}$$

where the target cell number (or concentration) *N_o_* can be derived from clinical data, *e.g*. SBRT of early stage lung cancer having *N_o_* from 10^4^ to 10^6^.^21^ Note that NTCP is defined in the same way as that of TCP because they share the same sigmoid shape of dose–responses but having different *N_o_* and *RS*. An example of the NTCP calculation for skin reactions will be provided in the result section.

BED at 50% TCP (or NTCP) is derived in Supplementary Material 1 as

,$${BED}_{50}\left(m,\gamma ,n,N_{o}\right)=\frac{ln\left[1-n+\frac{{\left(e^{\gamma D_{50}}-1+n\right)}^{m}}{n^{m-1}}\right]}{\gamma }$$

(8) $$D_{50}\left(m,\gamma ,n,N_{o}\right)=ln\left\{1+n\left[{\left(\frac{N_{o}}{ln\left(2\right)}\right)}^{\frac{1}{m}}-1\right]\right\}/\gamma$$

The total dose ${TD}_{50}=m∙D_{50}$ .

The steepness of a TCP (or NTCP) curve, Γ50, at 50% TCP (or NTCP) is^22^

(9) $$\Gamma_{50}=\frac{\delta TCP(orNTCP)}{\delta (TD)}{\left|{}_{50\%}=-0.0346\frac{1}{s}\frac{\delta S}{\delta D}\right|}_{D=D_{50}}=0.346\frac{\gamma }{n}Se^{\gamma D}|{}_{D=D_{50}}$$

The total dose to achieve 80% TCP or 20% NTCP can be estimated by TD_50_ +0.3/Γ_50_ for the tumour or TD_50_ - 0.3/Γ_50_ for the normal tissue, respectively.

The fraction dose for changing the course from D-Gy m-fractions to i-fractions with the same ending survival fraction of $S\left(i,D_{i}\right)=S\left(m,D\right)$ is given by

(10) $$D_{i}=\frac{1}{\gamma }ln\left\{1+n\left[\frac{1}{{S\left(D\right)}^{m/i}}-1\right]\right\}$$

## Results

**Figure 2.**
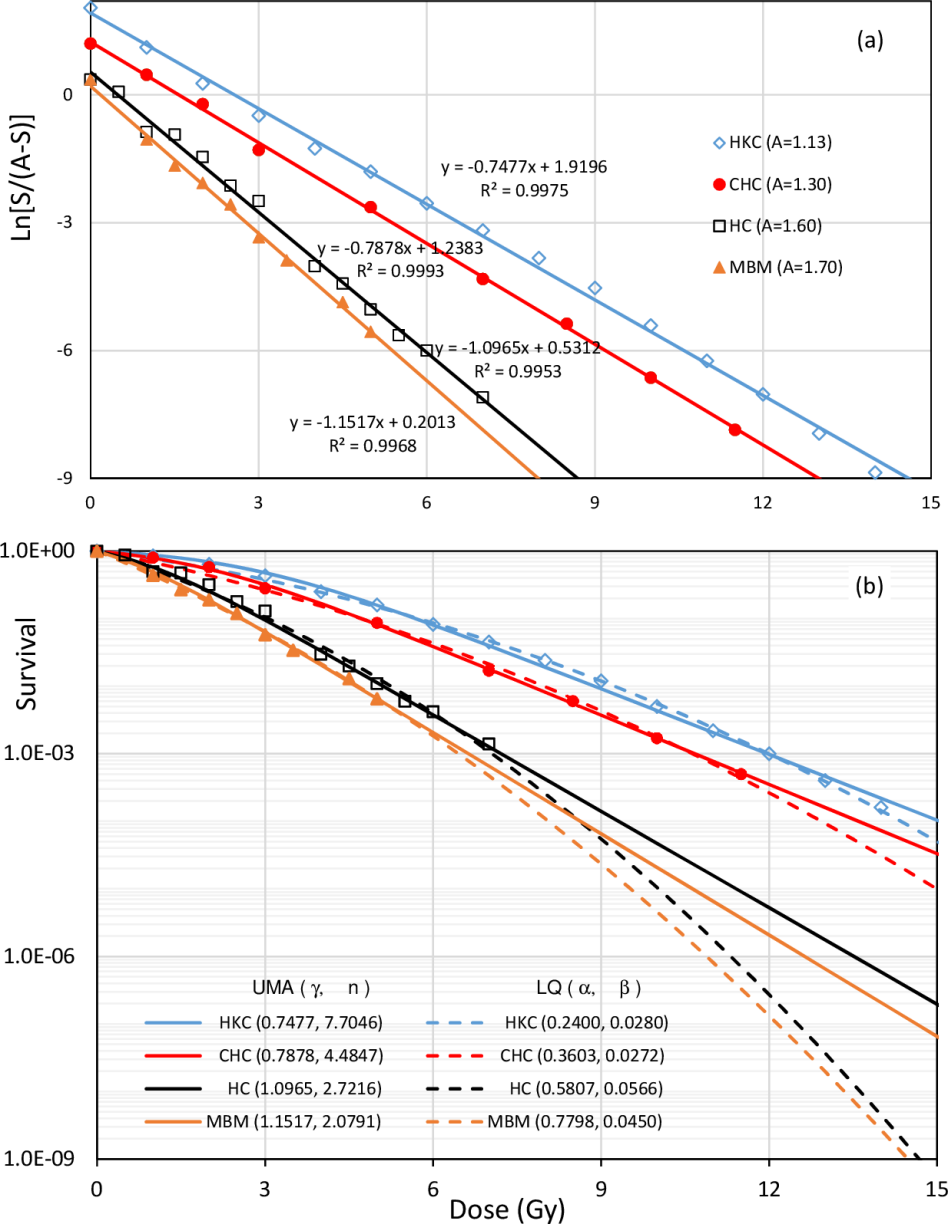
(**a**) Parameterised logit linear regression of HKC, CHC, HC and MBM for the UMA model with R^2^ >0.995. (**b**) Comparison between UMA (γ,n) and LQ (**a, b**) models for the same set of CSCs in logarithmic-linear plots where γ and α in Gy^−1^ while β in Gy^−2^. CHC, Chinese Hamster cell; CSC, cell survival curve; HC, human Hela cell; HKC, human kidney cell; LQ, linear quadratic; MBM, mouse bone marrow cell; UMA, unified multiactivation.

The UMA model fitting of CSCs for human kidney cells (HKC), Chinese Hamster cells (CHC), human Hela cells (HC) and mouse bone marrow cells (MBM) to X-rays with marked data points redrawn from Figs. 3.3 and 3.12 in Hall’s text book^23^ is presented in Figure 2. The modified logit function with listed parameter of A has transformed individual CSCs into straight lines and almost perfect fitting (R^2^ >0.99) as shown in Figure 2a. Both UMA and LQ models have explained over 99% variability of cell survivals by the dose in the experimental dose range, but the LQ model (dashed lines) and the UMA model (solid lines) differ systematically at the doses higher than the experimental range in Figure 2b. The fitting of other 33 CSCs of human cancer cell lines for typical sites of hypofractionated radiotherapy results γ from 0.1 to 2 Gy^−1^ and *n* from 0.2 to 60, respectively.^19^ The dose-independent *γ* and *n* as well as δ have confirmed the correct assumption in the derivation of Eq. (2).

**Figure 3.**
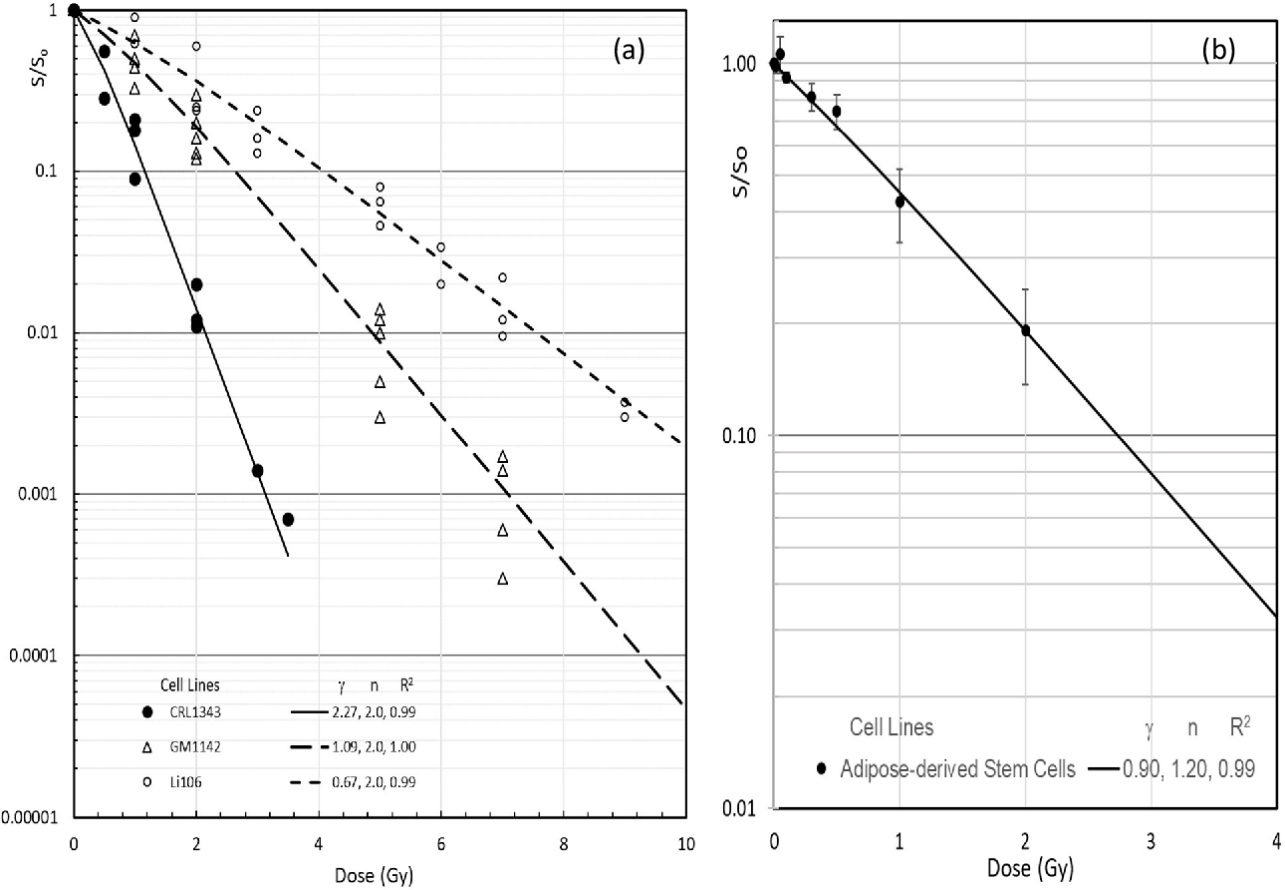
(**a**) UMA model results of X-ray survival curves of human skin fibroblasts: CRCL1343 from a patient with ataxia telangiectasia, GM1142 from a D-deletion retinoblastoma patient, and Li106 normal fibroblasts with marked data redrawn from Weichselbaum et al^26^. 3(b) UMA model results of a low dose X-ray survival curve of human adipose-derived stem cells from 10 donors for marked data with 1SD error bars redrawn from Schroder et al.^27^ UMA, unified multiactivation.

To anticipate the skin reactions to low doses from diagnostic X-ray imaging,^24,25^ the UMA model is applied to CSCs of human skin fibroblast cells by Weichselbaum et al^26^ and stem cells by Schröder et al^27^ as plots in Figure 3a and b, respectively. Skin intrinsic RS at zero dose defined by γ/*n* of the skin cell lines are from 0.34 to 1.14 Gy^−1^ that are even higher than the initial RS of 0.075/Gy to the squamous cell carcinoma.^19^ More importantly, skin RS increases with dose in diagnostic imaging (<0.1 Gy) as predicted by $RS\left(D\right)=\frac{\gamma }{\left[n+\left(1-n\right)\gamma D\right]}$ for any skin cell lines with *n* > 1 to X-rays. Different γ values of 0.67, 1.08 and 2.27 Gy^−1^ to the fibroblasts from a normal person, a patient with D-deletion retinoblastoma and a patient with ataxia telangiectasia, with the same of *n* = 2, indicate that the first-order reaction activation constant not the reaction ratio varies significantly among patients with genetic alterations. Recent imaging of human bulge cell morphology with molecular markers has greatly improved our understanding of the mechanism of hair loss.^28^ The temporary alopecia is likely come from the destruction of stem cells in the bulge of a hair follicle that responsible for hair shaft production but the number of the stem cells per hair follicle varies greatly with the stages of hair follicle cycle. During homeostasis, or over time after a wound, bulge cells diminish but can be regenerated or migrated for hair regeneration. Thus, the temporary alopecia occurred after taking an interventional neuroradiology procedure with a typical dose of 3 Gy^25^ can be predicted by the bulge stem cell survival (colonogenic assay^27^ ***S*** (3 Gy)=0.080 with measured γ = 0.90/Gy and *n* = 1.20). The number of stem cell clusters in the hair follicle bulge in homeostasis is estimated to be <5% of the peak value of ~600 from the K15 and CD34 double-positive marked stem cell population in the image figures of the paper by Cotsarelis.^28^ Use Eq. (7) for 30 cells (or clusters), the NTCP of temporary alopecia = NTCP(30,1, 3 Gy) is ~10%, that reasonably agrees with the clinical outcomes.^24,25^

Table 1 lists the predicted quantities for hypofractionated SBRT of early stage lung cancer by the UMA model of CSCs of human squamous cell carcinoma of the lung - SW1573 cells with 24 h delay plates (SW1573 24 h DP), four *in-vivo* cell lines of a large-cell carcinoma (HX147), a variant small cell carcinoma (HX149M), a classical small cell lung cancer (HC12) and an adenocarcinoma (HX144). The number of tumour cells *N_o_* = 10^4^, 10^5^, 10^6^ in the treated early stage lung cancer (affecting TCP, TD50 and G50 calculations) are selected based on a recent multiple institutions’ data and models’ study of Liu et al.^21^ The UAM model results clearly demonstrate followings: (1) the higher γ is, the lower *S*(10 Gy) gets since the first order reaction constant γ determines the cell survival at the high dose as $S\left(D\right)\approx ne^{\gamma D}$ ; (2) if fractional dose is within the straight portion of these CSCs, changing fraction number from 5 to 3 results the same dose of ~15 Gy per fraction for all of cell lines; (3) squamous cell carcinoma (SW1573) has the lowest RS(0) and BED_50_ but the highest GRS, EQD2, TCP and Γ_50_ mainly due to its large *n* or more repairing through the second-order reactions as $\delta =\frac{-\gamma }{N_{o}}\left(1-1/n\right)$ ; and (4) the large-cell carcinoma (HX147) has the lowest GRS (not the initial RS), EQD2, TCP and Γ_50_ but the highest S(10 Gy), D_50_ and BED_50_ due to its small γ and n. The large cells are ~2 times larger than the sizes of other cells and the same detectable gross tumour volume may contain ~10 times less number of the tumour cells. Having *N_o_* = 10^4^ instead of 10^5^, the TCP is increased from 26.5 to 87.6% and D_50_ is reduced from 10.5 to 8.9 Gy, respectively. TCP and BED_50_ for the large cell carcinoma (HX147) are still worse than that for SBRT of other types of the lung cancer. Thus, SBRT of early large cell carcinoma may require more dose than other types of the lung cancer. The impact of N_o_ on TCP, D_50_, BED_50_ and Γ_50_ are shown in Table 1. TCP exponentially decreases with *N_o_* as expressed in the formula (7). D_50_ (or TD_50_), BED_50_ and Γ_50_ are all increasing with N_o_ as expressed in formulas (8) and (9). N_o_ increase from 10^4^ to 10^6^ results significant changes of D_50_ and BED_50_ but less change on Γ_50_ for all cell lines shown in Table 1 for the 5-fractional SBRT of lung cancer cell lines. An important conclusion is that if the BED, specified to the cancer cell line and the selected course of treatment, is greater than the tumour BED_50_ by roughly 1/Γ50, complete tumour control is expected regardless to the tumour size (or number of cancer cells). Otherwise, the TCP would vary with the total number of cancer cells. All of values in the table are reasonable predictions based on the UMA modelling of preclinical CSCs. Thus, the new UMA model are useful in the design of a new treatment scheme for radiotherapy of cancer.

**Table 1.** Predicted quantities for *D* = 10 Gy x m=5 fraction SBRT of lung cancer cell lines

Cell lines	SW1573 24 h DP	HX147	HX149M	HX144	HC12
y in Gy^–1^	0.90	0.33	0.44	0.45	0.59
*n*	12.00	3.20	4.00	4.00	4.50
S(D)	0.0015	0.1058	0.0471	0.0412	0.0124
RS(O) in Gy^–1^	0.075	0.104	0.110	0.114	0.131
GRS in Gy^–1^	0.33	0.20	0.24	0.25	0.30
BED in Gy	38.97	35.50	37.27	37.68	39.75
EQD2 in Gy	185.45	86.57	100.96	101.23	108.50
TCP^*a*^	100.0%	87.6%	99.8%	99.9%	100.0%
TCP^*b*^	100.0%	26.5%	97.7%	98.8%	100.0%
TCP^*c*^	100.0%	0.0%	79.3%	88.9%	100.0%
D_50_^*a*^ in Gy	4.72	8.92	7.23	7.01	5.61
D50^*b*^ in Gy	5.30	10.42	8.38	8.12	6.47
D_50_^*b*^ in Gy	5.85	11.88	9.48	9.19	7.30
BED_50_^*a*^ in Gy	12.50	29.83	23.08	22.37	17.48
BED_50_^*b*^ in Gy	15.40	37.67	29.01	28.11	21.92
BED_50_^*c*^ in Gy	18.17	45.13	34.65	33.59	26.15
Γ_50_^*a*^ in Gy^–1^	0.27	0.10	0.14	0.14	0.18
Γ_50_^*b*^ in Gy^–1^	0.29	0.11	0.14	0.15	0.19
Γ_50_^*c*^ in Gy^–1^	0.29	0.11	0.15	0.15	0.19
D_3fx_ in Gy	14.83	14.67	14.69	14.74	14.99

BED, biologically effective dose; SBRT, stereotactic body radiotherapy; TCP, tumour control probability.

a These values are calculated with a total tumour cell number of N_o_ = 10^4^.

b These values are calculated with a total tumour cell number of N_o_ = 10^5^.

c These values are calculated with a total tumour cell number of N_o_ = 10^6^.

## Discussions

A simple unified formula of CSC using only two dose-independent parameters of γ and *n* is obtained based on an assumption that radiation-activated first- and second-order reactions through multiple cell dying processes (or CD pathways) apparently satisfy a logistic equation in the dose domain. The γ $\dot{D}$ is the first-order reaction rate constant while the n is the ratio of the first-order reaction rate to the total reaction rate. The UMA model and its two parameters are conceptually different from that of the traditional MT and LQ models. More importantly, the UMA model has almost perfectly fit typical CSCs over the entire dose range and completely resolved the catastrophes of the zero slope at zero dose from MT models and the bending curve at high dose from the LQ model.

The parameters γ and *n* could be precisely determined from a modified logit linear regression of *in-vivo* or close-to *in-vivo* CSC and the dose-independent parameters allows analytical predictions of dose–response quantities such as RS, GRS, EQD2, BED,^29^ TCP, NTCP, BED_50_, D_50_ and Γ_50_ at any dose levels. Thus, it could hold broad implications for clinical practice, particularly for alternative fractionation schemes or new therapeutic indications such as SBRT of multiple oligometastases^30^ by using the same biochemical mechanism with no modification of the parameters with low and high dose levels that differ from current practices using LQ or LQL models.^10–13,31^ The UMA model with a common mechanism for all dose levels is also useful for the risk assessments of low dose radiological procedures and radioprotection that still uses contradicted models.^15^

It is true that the UMA model have several facets to be explored such as the synergistic effects when combined with chemotherapy, hyperthermia, immunotherapy, radiosensitisers for the tumour cells, and/or radioprotectors for normal tissue. The dose–rate effects such as in ultra-high dose rate in FLASH radiation and blood supply changes during a course of the treatment may also influence the cell responses, biochemical reactions, or repairing processes. The UMA biochemical modeling of CD pathways is principally applicable to describe cell dying processes activated by other agents. But the continuous or pulsed activation by an agent in chemotherapy and long recovery time from hyperthermia may affect the dynamics of the cells of interest. Both theoretical and experimental investigations are required for the model extension.

## Conclusions

The successful UMA model of a CSC over the entire dose range reveals possibly a common mechanism - the first- and second-order reaction kinetics in cellular scale has integrally represented multiple compounding CD pathways activated by ionising radiation. Such a common mechanism allows us to analytically and reasonably quantify the clinical-interested doses and responses in radiotherapy and radiological imaging procedures by the UMA modeling of preclinical *in-vivo* CSCs.
